# Retinopathy of prematurity: A comprehensive risk analysis for prevention and prediction of disease

**DOI:** 10.1371/journal.pone.0171467

**Published:** 2017-02-14

**Authors:** Leah A. Owen, Margaux A. Morrison, Robert O. Hoffman, Bradley A. Yoder, Margaret M. DeAngelis

**Affiliations:** 1 Department of Ophthalmology and Visual Sciences, John Moran Eye Center, University of Utah School of Medicine, Salt Lake City, Utah, United States of America; 2 Department of Pediatrics, Division of Neonatology, University of Utah, Salt Lake City, Utah, United States of America; Massachusetts Eye & Ear Infirmary, Harvard Medical School, UNITED STATES

## Abstract

**Background:**

Retinopathy of prematurity (ROP) is a blinding morbidity of preterm infants. Our current screening criteria have remained unchanged since their inception and lack the ability to identify those at greatest risk.

**Objectives:**

We sought to comprehensively analyze numerous proposed maternal, infant, and environmental ROP risk variables in a robustly phenotyped population using logistic regression to determine the most predictive model for ROP development and severity. We further sought to determine the statistical interaction between significant ROP risk variables, which has not previously been done in the field of ROP. We hypothesize that our comprehensive analysis will allow for better identification of risk variables that independently correlate with ROP disease. Going forward, this may allow for improved infant risk stratification along a time continuum from prenatal to postnatal development, making prevention more feasible.

**Methods:**

We performed a retrospective cohort analysis of preterm infants referred for ROP screening in one neonatal intensive care unit from 2010–2015. The primary outcome measure was presence of ROP. Secondary outcome measures were ROP requiring treatment and severe ROP not clearly meeting current treatment criteria. Univariate, stepwise regression and statistical interaction analyses of 57 proposed ROP risk variables was performed to identify variables which were significantly associated with each outcome measure.

**Results:**

We identified 457 infants meeting our inclusion criteria. Within this cohort, numerous factors showed a significant individual association with our ROP outcome measures; however, stepwise regression analysis found the most predictive model for overall ROP risk included estimated gestational age, birth weight, the need for any surgery, and maternal magnesium prophylaxis. The corresponding Area Under the Curve (AUC) for this model was 0.8641, while the traditional model of gestational age and birth weight predicted ROP disease less well with an AUC of 0.8489. Development of severe ROP was best predicted by estimated gestational age (week), the need for any surgery and increased probability of death or moderate-severe BPD at 7 days. Finally, the model most predictive for type 1 ROP included estimated gestational age (week) and the presence of severe chronic lung disease. No significant statistical interaction was found between variables.

**Conclusions:**

Our work is unique as we report comprehensive analysis of the greatest number of proposed ROP risk variables to date in a robustly phenotyped population. We describe novel risk models for our ROP outcome measures and demonstrate independence of these variables using statistical modeling not previously applied to ROP. This may better allow for individual infant risk stratification and importantly mitigation of future risk.

## Introduction

Retinopathy of prematurity (ROP) is a blinding morbidity affecting preterm infants. It is a significant clinical problem and currently represents the leading preventable cause of childhood blindness worldwide. [1], [2] Prevelance varies by population though is estimated overall between 10–25% [3]–[5] and incidence between approximately 50–70% in infants weighing less than 1500 grams at the time of birth. [1], [6], [7] Most data indicate an increasing incidence of ROP disease as industrialized counteries report increased incidence by approximately 10 fold since the 1990’s.[8] ROP pathology is characterized by the presence of avascular retina and subsequent aberrant retinal neovascularization.[9] In the most severe stages of the disease retinal traction and detachment develop leading to permenant blindness.[9], [10] Recent work demonstrates the rates of severe, treatment-worthy, ROP rose from 1.7 to 14.8 per 1000 preterm infants between the years 1990 and 2011. [8] In fact, the National Eye Institute reports that approximately 1,100–15,00 infants will develop ROP requiring treatment each year in the US and approximately 400–600 will become legally blind from ROP. (https://nei.nih.gov/health/rop/rop) Thus, ROP is an increasing and significant clinical problem.

The clinical pathogenesis of ROP and its relationship with infant and environmental factors was first described in the 1940’s by Terry and colleagues. [11] Since that time, we have further clarified epidemiologic risk factors, such as early gestational age and low birth weight, which predispose to the development of ROP. [12]–[16] These continue to inform our screening protocols, which dictate absolute screening for infants born less than 30 weeks gestation or 1250 grams.[17], [18] However, these guidelines have remained largely unchanged since their original association with ROP disease and have low specificity. [6], [19], [20] Numerous epidemiologic studies have suggested additional risk factors for ROP disease and severity. [21]–[25] These include multiple birth, maternal preeclampsia, intrauterine growth restriction, mechanical ventilation, need for blood transfusion, the presence of patent ductus arteriosis, intraventricular hemorrhage, pulmonary insufficiency and male gender among others. [14]–[16], [20], [22], [23], [25]–[30] However, despite this work to date epidemiologic studies have not shifted our screening practice or increased screening sensitivity.

The reason for problematic incorporation of epidemiologic data into our screening paradigm is likely due to the variability in analysis approach, relatively small simultaneous factor analysis, changes in neonatology practice over time with regard to proposed risk factors such as oxygen supplementation, and small cohort size. For example, the majority of studies perform only univariate analysis, which does not account for the confounding effects of individual variable significance, chiefly known significant factors such as early gestational age. For those studies that do use a logistic regression or multivariate approach, most analyze only modest numbers of risk variables. [26], [31] Thus the analysis is not comprehensive with regard to suggested ROP risk variables. Finally, given the particularly low incidence of severe ROP as noted previously, sample size is often small for epidemiologic ROP studies. However, data compiled over a long duration, while it may increase sample size, includes infants treated very differently per neonatal intensive care unit (NICU) technology, target oxygen saturations, available interventions and medications. Thus, this also can introduce confounding.

Therefore, a knowledge gap remains, as despite the number of epidemiologic studies attempting to define ROP risk variables, we still cannot adequately predict infants at greatest risk for disease. [6], [32] More specific screening criteria are desperately needed however as our current screening exams are stressful for infants and caregivers and further, the majority of infants screened do not develop vision threatening disease. [20], [33] Thus, going forward we need to preserve our ability to identify all infants at risk for blinding disease, while better targeting our exams to appropriately exclude those at low risk. In our study we sought to address this knowledge gap through simultaneous analysis of the greatest number of proposed ROP risk variables to date in a roubstly phenotyped cohort to determine the most predictive models for ROP development and severity. We further sought to determine if there was a statistically significant interaction between significant variables, an analysis technique that has not previously been done in the field of ROP. Finally, we analyzed the predictive value of our models as compared with current screening criteria and a recently cited model. We hypothesize that by including a greater number of proposed ROP risk variables in a simulataneous anlaysis and within a well-phenotyped population receiving consistent NICU care, we would be better able to define individually singificant ROP risk factors and determine the most predictive risk models for ROP development and severity.

## Materials and methods

Study Cohort: We performed a retrospective cohort study of preterm infants at one neonatal intensive care unit between 2010 and 2015. Infants met inclusion criteria if they were deemed appropriate for ROP screening per the clinical NICU guidelines. For the NICU in our study this included infants born less than 30 weeks gestational age and 1250 grams, or infants who displayed an unstable medical course as determined by the attending neonatologist. The ladder is common practice thorughout the US to ensure all infants with any ROP risk receive appropriate screening; neonatologists making this clinical judgement were not involved in study analysis therefore study enrollment was not a confounding factor in their decision making. Infants were excluded from our study if they did not meet our NICU clinical criteria for ROP screening. We did not set an *a priori* study size but rather designed our study to include years during which neonatology care practice and importantly oxygen saturation standards were uniform to remove these potentially confounding factors. All data collection was approved by the Institiutional Human Subjects Committees at the University of Utah, adhered to the Declariation of Helsinki, and was compliant with the Health Insurance Portability and accountability Act (HIPAA). Data were collected with waiver of consent for de-identified data. No authors have a conflict of interest.

Data Collection: Infant and environmental risk factor data were collected for the duration of infant NICU stay. This duration is sufficient to document all collected indices. Maternal data were collected retrospectively at the time of infant enrollment. Data for a total of 57 indices were collected and analyzed. (Table 1) These risk factors were chosen through collaboration with pediatric ophthalmology and neonatology physicians who specialize in the diagnosis and treatment of ROP. In brief, factors were chosen based on the preponderance of literature suggesting their role in ROP and specialist recommendation. [23], [24], [26], [28] The probability of death or moderate-severe bronchopulmonary dysplasia at 7 and 14 days is a clinically calculated indices commonly assessed by neonatologists, as referenced. [34]

**Table 1:** Proposed ROP risk variables. Included are all tested ROP risk variables and percentage representation in each outcome measure.

	No ROP (n = 217)		Any ROP (n = 240)		Type 1 ROP (n = 33)		Severe ROP (n = 53)	
**MATERNAL RISK FACTOR**								
**Assessed ROP Risk Variable**	**No./Ave.**	**Percent/Range**	**No./Ave.**	**Percent/Range**	**No./Ave.**	**Percent/Range**	**No./Ave.**	**Percent/Range**
Antenatal Betamethasone doses	1.9	0–4	1.7	0–4	1.4	0–2	1.6	0–5
Chorioamnionitis	37	17.10%	49	20.40%	16	48.50%	12	22.60%
Chronic Maternal Hypertension	15	6.90%	15	6.30%	2	6.10%	3	5.70%
Maternal age in years	28.4	14–50	28	15–58	27.1	16–44	27.3	16–44
Maternal Magnesium prophylaxis	150	69.10%	189	78.80%	26	78.80%	43	81.10%
Placental Abruption	38	17.50%	38	15.80%	3	9.10%	4	7.50%
Pre-eclampsia	28	12.90%	35	14.60%	3	9.10%	5	9.40%
Preterm Rupture of Membranes (ROM)	95	43.80%	94	39.20%	14	42.40%	21	39.60%
ROM > 24 hours	62	28.60%	61	25.40%	7	21.20%	13	24.50%
ROM > 7 days	34	15.70%	38	15.80%	4	12.10%	7	13.20%
**Birth Season**								
Winter	49	22.60%	57	23.80%	11	33.30%	17	32.10%
Spring	67	30.90%	69	28.80%	4	12.10%	9	17.00%
Summer	58	26.70%	72	30.00%	9	27.30%	15	28.30%
Fall	43	19.80%	42	17.50%	9	27.30%	12	22.60%
**Maternal Diabetes**								
Gestational	10	4.60%	5	2.10%	0	0.00%	0	0.00%
Prior	5	2.30%	7	2.90%	1	3.00%	1	1.90%
**Maternal Race**								
White race	136	62.70%	160	66.70%	18	54.50%	33	62.30%
Black race	4	1.80%	10	4.20%	0	0.00%	0	0.00%
Hispanic race	54	24.90%	47	19.60%	10	30.30%	15	28.30%
Asian or other race	23	10.60%	23	9.60%	5	15.20%	5	9.40%
**INFANT RISK FACTOR**								
Any Intraventricular Hemorrhage (IVH)	37	17.10%	94	39.20%	18	54.50%	28	52.80%
Highest Intraventricular Hemorrhage	0.3	0–4	1.0	0–4	1.2	0–4	1.3	0–4
APGAR Score at 1 min	4.7	1–9	4	0–9	3.3	0–8	3.5	0–8
APGAR Score at 5 min	7	1–9	6.3	1–9	6	1–9	6.1	1–9
APGAR Score < 5 at 5 min	19	8.80%	46	19.20%	7	21.20%	10	18.90%
Birth weight in grams	1140.3	410–1490	837.7	410–2520	689.3	410–910	710	410–1110
Birth weight <1000 grams	62	28.57%	188	78.33%	33	100.00%	52	98.11%
Birth weight <1250 grams	145	66.82%	227	94.58%	33	100.00%	53	100.00%
Chronic Lung Disease	67	31.00%	160	66.70%	27	81.80%	45	84.90%
Severe chronic lung disease	27	12.50%	96	40.17%	22	68.75%	32	61.54%
Fetal Anomoly	4	1.80%	8	3.33%	0	0.00%	0	0.00%
Focal Ileal perforation	1	0.04%	10	4.20%	4	12.10%	5	9.40%
Gestational Age in weeks	28.31	24.00–29.86	26.15	23.00–29.86	24.76	23.00–26.29	24.95	23.00–29.71
Intrauterine growth restriction (IUGR)	26	12.00%	23	9.60%	2	6.10%	5	9.40%
Male gender	117	53.92%	125	52.08%	16	48.48%	27	50.94%
Multiple Birth Gestation	75	34.60%	70	29.20%	7	21.20%	13	24.50%
Necrotizing Enterocolitis (NEC)	4	1.80%	12	5.00%	4	12.10%	6	11.30%
NEC requiring surgical treatment	22	10.10%	34	14.20%	6	18.20%	8	15.10%
Patent Ductus Arteriosus (PDA)	93	42.90%	177	73.75%	30	90.91%	47	88.68%
PDA treated medically	35	16.10%	97	40.40%	18	54.50%	31	58.50%
PDA treated surgically with ligation	7	3.20%	48	20.00%	13	39.40%	20	37.70%
Probability of death or moderate-severe BPD at 14 days	25.1	1.0–93.6	59.8	1.8–97.8	76.2	20.9–92.1	77	16.0–94.8
Probability of death or moderate-severe BPD at 7 days	24.6	1.0–93.8	59.6	2.0–96.3	78.6	30.4–94.4	77.6	30.4–94.4
**ENVIRONMENTAL RISK FACTOR**								
Any surgical procedure	26	12.00%	113	47.50%	33	100.00%	44	83.00%
Blood transfusion	88	43.30%	200	85.80%	32	100.00%	51	98.10%
Caffeine administration	96	44.00%	109	45.40%	13	39.40%	22	41.50%
Age Caffeine administered	14.3	1.0–240.0	34.8	1.0–720.0	38.2	1.0–408.0	41.5	1.0–408.0
Days on a ventilator	6.1	0.0–81.0	29.4	0.0–120.0	44.3	0.1–93.0	42.7	0.1–98.0
Days on continuous positive airway pressure (CPAP)	9	0.0–59.0	18.2	0.0–65.0	22.2	0.0–58.0	20.8	0.0–58.0
Days on high flow nasal cannula oxygen	12.4	0.0–111.0	17.5	0.0–100.0	23.2	1.0–80.0	20.1	0.0–80.0
Delivery by C-Section	157	72.40%	164	68.30%	17	51.50%	28	52.80%
Dexamethasone administration	29	13.40%	111	46.40%	20	62.50%	34	65.40%
Age Dexamethasone administered	43.7	1.0–177.0	33.4	6.0–97.0	38	9.0–96.0	34.3	7.0–96.0
Dopamine administration < 72 hours of life	22	10.10%	83	34.60%	15	45.50%	22	41.50%
Hydrocortisone therapy < 72 hours of life	16	8.60%	73	30.42%	16	48.48%	21	39.62%
Inhaled nitric oxide (iNO)	16	8.60%	22	15.10%	2	14.30%	5	19.20%
Intubation	175	80.60%	225	93.80%	33	100.00%	53	100.00%
Age at intubation	99.2	1.0–1536.0	48.6	0.5–6120.0	9	1.0–89.0	8	1.0–89.0
Resuscitation need	3.3	1–5	3.8	1–5	4.2	3–5	4.1	3–5
Surfactant administration	153	70.50%	219	91.30%	33	100.00%	53	100.00%
Total days on oxygen	35.4	0.0–124.0	55.8	0.0–240.0	75.2	0.0–161.0	75.6	0.0–240.0
**Birth Position**								
Breech	77	35.48%	103	42.92%	15	45.45%	24	45.28%
Normal	0	0.00%	1	0.42%	0	0.00%	0	0.00%
Other	0	0.00%	1	0.42%	0	0.00%	0	0.00%
Transverse	18	8.29%	9	3.75%	1	3.03%	1	1.89%
Unknown	1	0.46%	2	0.83%	1	3.03%	1	1.89%
Vertex	121	55.76%	124	51.67%	16	48.48%	27	50.94%

APGAR: Appearance, Pulse, Grimace, Activity, Respiration; BPD: Bronchopulmonary Dysplasia

Outcome Measure Phenotype: All infant ROP phenotypes were assessed by pediatric ophthalmologists during the course of normal clinical care. The primary outcome measure was development of ROP. Secondary outcome measures were 1) development of ROP disease requiring treatment termed type 1 ROP as defined by the Early Treatment of ROP study. [7] and 2) development of severe ROP which we defined as zone 1 or 2 stage 3 ROP or greater in the worse eye. We used the universally accepted Revised International Classification of ROP guidelines to define the location and extent of disease within the retina.[35], [36] These criteria represent consensus guidelines for describing where the ROP disease occurs within the retina, whether it is close to the area of central vision and therefore more concerning (zone 1) or in a more peripheral portion of the retina (zone 3). These criterion also allow for standardized discussion of disease severity by stage. For example, stage 1 is the least severe form of ROP, correlating with a demarcation line between vascular and avascular retina and stage 5 is the most severe, correlating with total retinal detachment. Notably for our study, stage three as included in our “severe ROP” outcome measure, is the first stage where aberrant neovascularization is found. Our delineation of severe ROP therefore includes infants with eye disease that is worrisome by clinical standards, but does not have an absolute indication for treatment by current standards. For infants requiring multiple exams, ROP zone and stage for analysis was from the highest severity examination in the worse eye as is standard reporting practice for ROP studies. Importantly, these data were collected seperately from the time of clinical exam, therefore screening clinicians were not influenced by patient study inclusion.

Statistical Analysis: Proposed ROP risk variables were tested for association with each ROP outcome measure using logistic regression in SAS (v9.3, SAS Institute Inc., Cary, NC). Nominal significance was considered a p-value of <0.05 and estimates were reported as odds ratios with 95% confidence intervals. Subsequently, stepwise logistic regression was performed in order to determine the most predictive model for ROP. Specifically, those variables showing statistical significance at p <.05 in the single factor analysis were included in the stepwise regression model. Subsequently, each variable is added one by one to the model at a significance level of p≤ .1. After the addition of each variable, variables already in the model are removed if they do not remain significant at p < .05.

To test the effectiveness of our model, we created receiver operating characteristic (ROC) curves using SAS to compare the stepwise regression model from the current study to both the traditional ROP risk model which informs our current screening criteria, namely gestational age less than 30 weeks and birth weight less than 1250 grams, and the model recently proposed by Slidsborg et al. to be a standard model. [26]

Additionally, we performed interaction analysis to test for possible interactions within the current dataset. In order to control for potential confounders that may interfere with the interpretation of the interaction model, tests for interactions between the risk factors were performed using interaction terms as well as main effects in the logistic regression model, following the methodology proposed by Keller.[37] Specifically, factors that were shown to be nominally significantly associated with each ROP subtype (p < .05) were included in the model along with their corresponding interaction terms.

## Results

546 infants were initally identified for inclusion in our study; 66 infants died prior to the first eye examination and 23 did not meet screening criteria due to birth weight greater than 1250 grams; 457 infants met inclusion criteria. Data were collected for the duration of the infant’s NICU stay which was on average 10 weeks from the time of first eye exam. Baseline characteristics relative to each proposed ROP risk variable for our cohort of 457 infants, as represented in Table 1, show a fairly equal male and female population with a cohort average gestational age of 27.12 weeks and weight of 987 grams. Maternal race analysis demonstrated a predominantly Caucasian population, consistent with the demographic of Utah. We found that the overall ROP incidence proportion in our population was 47.5%, which decreased to 12% for severe ROP, and 7.2% for type 1 ROP. This is consistent with the published incidence of disease among preterm infants in the US. [1]

Univariate analysis was performed for all 57 indices, which have been suggested in the literature to contribute to ROP risk, though have not been comprehensively analyzed. A nominally statistically significant (p< .05) relationship was found between a number of factors and our ROP outcome measures. (Table 2) Importantly, we found factors historically known to influence ROP risk such as gestational age and birth weight to demonstrate a significant association for all outcome measures in the univariate analysis. [6]

**Table 2:** Univariate risk dactor analysis: Each of the 57 proposed ROP risk variables were assess for statistical association with each ROP outcome measure. Maternal, infant and environmental risk variables found to be nominally significant (p<0.5) for each outcome measure are highlighted in red.

	Any ROP, n = 240 vs. No ROP			Type 1 ROP, n = 33 vs. No ROP			Severe ROP, n = 53 vs. No ROP		
Risk Factor	Odds Ratio	95% CI	p value	Odds Ratio	95% CI	p value	Odds Ratio	95% CI	p value
Antenatal Betamethasone doses	2.88	0.61–0.94	0.012	0.50	0.32–0.78	0.002	0.65	0.46–0.92	0.015
Chorioamnionitis	3.88	0.78–2.00	0.358	1.08	0.42–2.80	0.873	1.42	0.68–2.97	0.345
Chronic maternal hypertension	0.90	0.43–1.88	0.775	0.87	0.19–3.99	0.856	0.81	0.23–2.90	0.744
Maternal age in years	14.88	0.96–1.02	0.537	0.97	0.91–1.03	0.271	0.97	0.93–1.02	0.262
Maternal Magnesium Prophylaxis	13.88	1.09–2.53	0.019	1.66	0.69–4.01	0.261	1.92	0.91–4.05	0.086
Placental Abruption	0.89	0.54–1.45	0.630	0.47	0.14–1.62	0.233	0.39	0.13–1.13	0.082
Pre-eclampsia	1.15	0.68–1.97	0.603	0.68	0.19–2.36	0.538	0.70	0.26–1.92	0.492
Preterm rupture of membranes (ROM)	0.83	0.57–1.20	0.318	0.95	0.45–1.99	0.884	0.84	0.46–1.56	0.584
ROM > 24 hours	18.88	0.56–1.29	0.447	0.67	0.28–1.63	0.381	0.81	0.41–1.62	0.556
ROM > 7 days	19.88	0.61–1.68	0.962	0.74	0.25–2.25	0.598	0.82	0.34–1.97	0.655
**Birth Season**									
Fall vs. Summer	0.79	0.46–1.36	0.518	1.35	0.49–3.68	0.257	1.08	0.46–2.54	0.598
Spring vs. Summer	0.83	0.51–1.34	0.679	0.39	0.11–1.32	0.033	0.52	0.21–1.28	0.049
Winter vs. Summer	0.94	0.56–1.57	0.730	1.45	0.55–3.78	0.154	1.34	0.61–2.96	0.150
**Maternal Diabetes**									
Gestational vs. None	0.44	0.15–1.32	0.139	<0.01	<0.01->999.99	0.973	<0.01	<0.01->999.99	0.968
Prior vs. None	1.24	0.39–3.97	0.337	1.26	0.14–11.16	0.972	0.78	0.09–6.80	0.969
Maternal race	0.91	0.77–1.08	0.274	1.18	0.87–1.61	0.290	1.01	0.78–1.32	0.931
Any Intraventricular Hemorrhage	3.13	2.02–4.86	<.001	5.84	2.7–12.62	<.001	5.45	2.86–10.39	<.001
Highest Intraventricular hemorrhage	11.88	1.38–2.03	<.001	1.95	1.45–2.63	<.001	1.99	1.53–2.58	<.001
APGAR Score at 1 min	0.88	0.82–0.95	0.002	0.78	0.66–0.92	0.003	0.81	0.71–0.92	0.002
APGAR Score at 5 mins	1.88	0.73–0.90	<.001	0.77	0.64–0.92	0.005	0.78	0.67–0.92	0.002
APGAR Score < 5 at 5 min	2.47	1.40–4.37	0.002	2.81	1.08–7.32	0.035	2.42	1.05–5.58	0.037
Birth weight in grams	1.00	1.00–1.00	<.001	0.99	0.99–0.99	<.001	0.99	0.99–0.99	<.001
Birth weight <1000 grams	9.04	5.91–13.83	<.001	>999.99	<0.01->999.99	0.925	129.99	17.58–960.86	<.001
Birth weight <1250 grams	8.67	4.63–16.21	<.001	>999.99	<0.01->999.99	0.952	>999.99	<0.01->999.99	0.942
Chronic Lung Disease	4.45	3.00–6.60	<.001	10.01	3.95–25.37	<.001	12.51	5.59–27.99	<.001
Severe chronic lung disease	4.70	2.91–7.59	<.001	15.40	6.59–36.01	<.001	11.20	5.62–22.30	<.002
Fetal anomaly	7.88	0.61–5.87	0.268	<0.01	<0.01->999.99	0.984	<0.01	<0.01->999.99	0.986
Focal Ileal Perforation	9.39	1.19–73.98	0.033	29.79	3.22–275.77	0.003	22.50	2.57–197.00	0.005
Estimated gestational age	0.39	0.33–0.46	<.001	0.09	0.04–0.21	<.001	0.15	0.08–0.25	<.001
Estimated Gestational Age (week)	0.40	0.34–0.47	<.001	0.09	0.04–0.22	<.001	0.15	0.09–0.26	<.001
IUGR	0.78	0.43–1.41	0.408	0.47	0.11–2.10	0.325	0.77	0.28–2.10	0.603
Female Gender	1.08	0.75–1.56	0.695	1.24	0.60–2.59	0.561	1.13	0.62–2.06	0.697
Multiple Birth Gestation	0.78	0.53–1.16	0.216	0.51	0.21–1.23	0.134	0.62	0.31–1.22	0.165
Necrotizing Enterocolitis (NEC)	1.46	0.83–2.59	0.192	1.97	0.73–5.29	0.179	1.58	0.66–3.77	0.307
NEC requiring surgical treatment	15.88	0.89–8.82	0.078	7.35	1.74–30.98	0.007	6.80	1.85–25.04	0.004
Patent Ductus Arteriosus (PDA)	16.88	2.53–5.55	<.001	13.33	3.95–45.02	<.001	10.44	4.28–25.46	<.001
PDA treated medically	17.88	2.26–5.50	<.001	6.24	2.88–13.54	<.001	7.33	3.81–14.11	<.001
PDA treated surgically with ligation	7.50	3.31–16.97	<.001	19.50	6.98–54.46	<.001	18.18	7.13–46.33	<.001
Probability of death or moderate-severe BPD at 14 days	1.05	1.04–1.06	<.001	1.09	1.06–1.12	<.001	1.08	1.06–1.11	<.001
Probability of death or moderate-severe BPD at 7 days	1.05	1.04–1.06	<.001	1.09	1.06–1.12	<.001	1.09	1.06–1.11	<.001
Any surgical procedure	6.64	4.10–10.76	<.001	>999.99	<0.01->999.99	0.943	35.91	15.73–82.01	<.001
Blood transfusion	7.92	4.99–12.56	<.001	>999.99	<0.01->999.99	0.936	66.65	9.03–491.69	<.001
Caffeine administration	1.05	0.73–1.52	0.801	0.82	0.39–1.73	0.601	0.89	0.49–1.64	0.719
Age Caffeine administered	1.00	1.00–1.01	0.110	1.01	1.00–1.01	0.163	1.01	1.00–1.01	0.085
Days on a ventilator	1.07	1.05–1.08	<.001	1.10	1.07–1.13	<.001	1.10	1.07–1.12	<.001
Days on CPAP	1.05	1.04–1.07	<.001	1.06	1.04–1.09	<.001	1.06	1.04–1.08	<.003
Days on high flow nasal cannula oxygen	1.02	1.01–1.03	0.002	1.03	1.01–1.05	0.002	1.02	1.01–1.04	0.006
Delivery by C-Section	4.88	0.55–1.23	0.349	0.41	0.19–0.86	0.018	0.43	0.23–0.79	0.007
Dexamethasone administration	5.88	3.53–8.96	<.001	10.81	4.78–24.42	<.001	12.24	6.13–24.46	<.001
Age Dexamethasone administered	0.98	0.97–1.00	0.053	0.99	0.98–1.01	0.537	0.99	0.97–1.01	0.216
Dopamine administration < 72 hours	6.88	2.80–7.84	<.001	7.39	3.27–16.69	<.001	6.29	3.12–12.69	<.001
Hydrocortisone therapy < 72 hours of life	5.49	3.08–9.79	<.001	11.82	5.05–27.71	<.001	8.24	3.90–17.45	<.001
Inhaled nitric oxide (iNO)	9.88	0.95–3.74	0.069	1.77	0.36–8.62	0.479	2.53	0.84–7.61	0.099
Intubation	10.88	1.93–6.70	<.001	>999.99	<0.01->999.99	0.963	>999.99	<0.01->999.99	0.956
Age at intubation	1.00	1.00–1.00	0.235	0.98	0.95–1.01	0.181	0.97	0.94–1.00	0.067
Resuscitation need	2.21	1.72–2.83	<.001	5.05	2.43–10.47	<.001	4.98	2.62–9.49	<.001
Surfactant administration	4.36	2.56–7.44	<.001	>999.99	<0.01->999.99	0.955	>999.99	<0.01->999.99	0.945
Total days on oxygen	1.01	1.01–1.02	<.001	1.03	1.02–1.04	<.001	1.02	1.01–1.03	<.001
**Birth Position**									
Breech vs. Normal	<0.01	<0.01->999.99	0.980	n/a	n/a	n/a	n/a	n/a	n/a
Transverse vs. Normal	<0.01	<0.01->999.99	0.976	n/a	n/a	n/a	n/a	n/a	n/a
Unknown vs. Normal	<0.01	<0.01->999.99	0.982	n/a	n/a	n/a	n/a	n/a	n/a
Vertex vs. Normal	<0.01	<0.01->999.99	0.980	n/a	n/a	n/a	n/a	n/a	n/a
Other vs. Normal	1.00	<0.01->999.99	0.989	n/a	n/a	n/a	n/a	n/a	n/a

In order to determine the most predictive model of ROP, we performed stepwise regression analysis. When comparing infants in our cohort who developed any form of ROP to those who did not, we found the most predictive model of overall ROP risk included estimated gestational age, birth weight, the need for any surgery, and maternal magnesium prophylaxis (Table 3). The corresponding concordance index, c, (an estimate of the area under the receiver operating characteristic (ROC) curve) for this model was 0.870. Development of severe ROP was best predicted by estimated gestational age (week), the need for any surgery and increased probability of death or moderate-severe BPD at 7 days (c = .978). (Table 3) Finally, the model most predictive for type 1 ROP included estimated gestational age (week) and severe chronic lung disease when comparing to those infants without ROP(c = .990). (Table 3)

**Table 3:** Results from stepwise regression analysis. Proposed ROP risk variables found to have nominal significance on univariate analysis were used in stepwise regression analysis for each outcome measure to determine the most predictive model for each ROP outcome measure.

	**Any ROP, n = 240 vs. No ROP**		
**Risk Factor**	**Adjusted Odds Ratio**	**95% CI**	**p value**
Estimated gestational age	0.487	(0.370–0.642)	< .001
Birth weight	0.998	(0.996–0.999)	0.003
Any surgery	2.891	(1.287–6.494)	0.010
Maternal magnesium prophylaxis	0.493	(0.246–0.989)	0.046
	**Severe ROP, n = 53 vs. No ROP**		
**Risk Factor**	**Adjusted Odds Ratio**	**95% CI**	**p value**
Any surgery	6.414	1.263–32.577	0.025
Risk of Bronchopulmonary dysplasia at 7 days of life	1.038	1.003–1.074	0.035
Estimated gestational age (week)	0.341	0.184–0.633	< .001
	**Type 1 ROP, n = 33 vs. No ROP**		
**Risk Factor**	**Adjusted Odds Ratio**	**95% CI**	**p value**
Severe chronic lung disease	10.116	1.317–77.73	0.026
Estimated gestational age (week)	0.099	0.03–0.325	< .001

**Fig 1:**
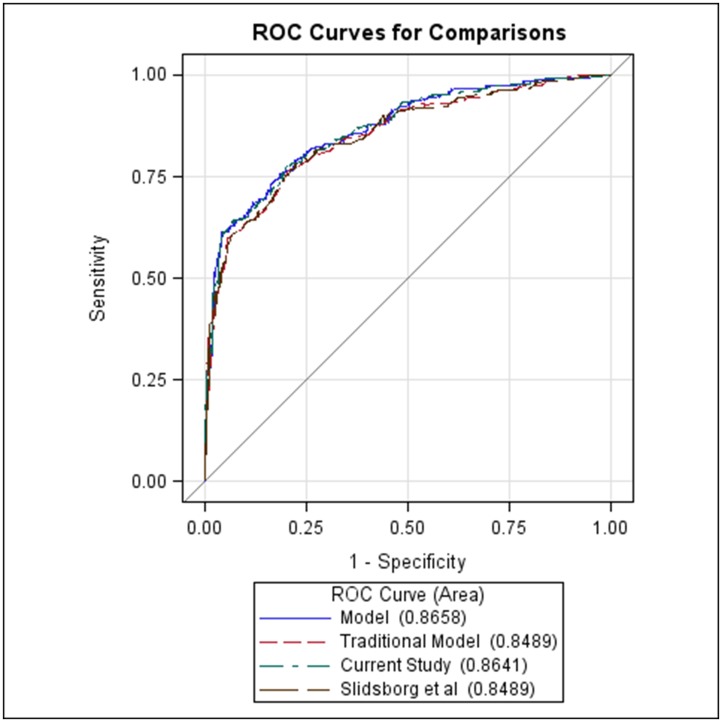
ROC curves comparing ROP prediction models. “Traditional Model” includes estimated gestational age and birth weight less than 1250 grams. “Current Study” includes estimated gestational age, birth weight, the need for any surgery, and magnesium. “Slidsborg et al” includes the traditional model used in the Slidsborg paper: Small for gestational age, estimated gestational age, gender, and multiple gestation. “Model” includes all variables used across each of the 3 comparison models.

ROC curves were created for the stepwise regression model from the current study as compared to the traditional ROP risk model and standard model used by Slidsborg et al. (Fig 1). The “Traditional Model” used included the variables estimated gestational age and birth weight less than 1250 grams as these are the classically accepted ROP risk variables currently utilized to inform infant ROP risk and screening. The “Current Study” included the results from our stepwise regression analysis of any ROP: estimated gestational age, birth weight, the need for any surgery, and maternal magnesium prophylaxis. The “Slidsborg et al” model included the traditional model used in the Slidsborg paper: Small for gestational age, estimated gestational age, gender, and multiple gestation. For the purposes of this study, the variable intrauterine growth restriction was used to represent small for gestational age as this is the corollary clinical parameter used in our NICU. When comparing the area under the curve (AUC) for each of these models, the stepwise regression model from the current study gave the greatest AUC, 0.8641, while the traditional model and the traditional model used by Slidsborg et al. provided the same AUC, 0.8489. Interestingly, the model that produced the greatest AUC was that using all variables included in each of the three models: estimated gestational age, birth weight, birth weight less than 1250 grams, intrauterine growth restriction, gender, multiple gestation, the need for any surgery, and maternal magnesium prophylaxis (AUC = 0.8658).

Finally, we sought to determine the statistical interaction between ROP risk variables for each disease phenotype. Keller et al. have demonstrated this technique as a way to determine interaction between disease risk variables.[37] However, no significant statistical interaction was seen between variables tested in our model.

## Discussion

ROP is an important clinical problem that if undetected can result in permanent, life-long blindness. With improved preterm infant survival, not only in the developed but also the developing world, the scope of the problem is increasing.[1] Our present screening guidelines are largely unchanged since their inception in the 1990’s and [32] most sources estimate that only 5–10% of infants screened under these guidelines will develop vision-threatening disease.[20], [33] Therefore, while the sensitivity is uniformly high for current screening guidelines, the specificity is low.[20] Work to model risk and better predict ROP has been complicated by the multifactorial nature of this disease.[32] Further, current statistical modeling of ROP risk has shown variable sensitivity across ethnic populations and does not account for broad sources of ROP risk. [38], [39]

Herein, we provide the most comprehensive analysis to date of proposed maternal, infant, and environmental ROP risk variables. Using this approach, we find that most of the factors assessed, while significant in a univariate analysis, fail to show significance in stepwise regression analysis. Consistent with the majority of literature, early gestational age demonstrates significance for all of our ROP outcome measures.[6], [22] Individual significance of early birth as compared with low birth weight is supported by other studies. For example, Woo et al., have shown that in twin gestations discordant for gestational weight, gestational age was a better predictor of ROP disease. [40] However, a number of factors postulated to be significant in current literature by univariate and multivariate analysis such as pre-eclampsia, patent ductus arteriosus, maternal age, blood transfusion, male gender, and multiple birth, do not show individual disease significance when corrected for confounding significance in our population.[26]

The variability of our findings compared with reported work speaks to both the strengths and weaknesses of our study. Strengths of our study include our comprehensive analysis of the most numerous set of proposed ROP risk variables published to date. Therefore, when accounting for a larger number of variables we may be better able to more accurately control for confounding effects of individual variables and better determine those variables that are independently predictive of ROP disease. Further, we report a robustly phenotyped cohort with uniform clinical care over a 5-year time period to further strengthen our ability to identify individually significant ROP risk variables. As is noted by Slidsborg et al., variability in clinical NICU practice with regard to oxygen administration etc as well as clinical data collection confounds analysis of variable contribution to ROP risk and limits inclusion or analysis of continuous variables.[26]

Several factors however, may limit the application of our findings and require further study. In an effort to include only infants with uniform neonatal care and thus minimize this important confounder, we limit our sample size. This limits the number of observations for some of our risk variables, most notably for the less common and most severe, Type 1 ROP outcome measure and may introduce a type 2 statistical error. However, increased sample size does not insure increased observations, particularly with respect to our more severe ROP outcomes. In fact, assessment of a more homogeneous population will counter this limitation and reduce the likelihood of spurious association.[39] The dichotomy between sample size and homogeneity of sample population is common in ROP literature, as often maximizing sample size requires inclusion of infants with dissimilar perinatal care. As these interventions comprise a significant proportion of our ROP risk variables, and indeed those currently suggested in the literature, we felt it most important to include only infants with uniform neonatal care. We are additionally limited by the demographic of our NICU setting, which is predominantly Caucasian. Racial differences have been demonstrated in the sensitivity and specificity of other proposed ROP risk models and possibly in ROP incidence and therefore, our findings should be replicated in a more diverse population.[41] Finally, the predictive utility of our model versus other models is an imperfect comparison as the sample populations are different in our cohort versus the comparison Danish population. Therefore, there may be elements of our population that better predispose to prediction using our model versus the proposed Danish model; this is not a parameter that can be fully controlled and should be noted. Overall however, we argue that our presentation of a smaller cohort with more uniform clinical care and robust phenotyping offers valuable insight into ROP risk. Certainly replication of our findings in a larger, more racially diverse, population will allow for clarity on this point.

In addition to our stepwise regression analyses, we also conducted a robust interaction analysis. This alternate statistical modeling system takes into account the effects of confounding variables in a slightly different manner in order to determine the degree of interaction between variables. This has not been done previously to characterize ROP risk. Using this methodology, we found no significant statistical interactions between the variables considered. Thus, the risk factors we identified are independent of one another, and by this analysis, confer independent risk.

In conclusion, better understanding of factors important for ROP development is critical for improved infant risk stratification and blindness prevention. Our approach is unique in the breadth and number of covariates queried and the degree of statistical modeling employed.[20], [28] While our findings reiterate the accepted importance of early birth and low birth weight to ROP risk, we also find that additional consideration of need for any surgery and maternal magnesium prophylaxis create the most predictive model for development of ROP. When considering the area under the curve in our ROC analysis our proposed model better predicts ROP development than the parameters used currently to stratify infant ROP risk.

Our work has also defined novel risk variables for severe ROP disease. Specifically, we describe risk of severe ROP disease with increased probability of death or moderate-severe BPD at 7 days. This is an emerging parameter used by neonatologists to assess primarily lung outcomes in preterm infants, though our work suggests utility for severe ROP prediction as well.[34] Future work will allow us to understand the predictive value of the models we describe, and also importantly, whether modulation of risk variables, when possible, can be exploited to alter the ROP development or severity. For example, infants identified as at risk for ROP development using our model may benefit from delay of elective surgical procedures until they reach a gestational age not associated with ROP development.[32] Certainly future work is necessary to clarify the validity and potential role of the models generated herein to ROP risk stratification.
